# Developing a sociocultural framework of compliance: an exploration of factors related to the use of early warning systems among acute care clinicians

**DOI:** 10.1186/s12913-020-05615-6

**Published:** 2020-08-11

**Authors:** Tracy Flenady, Trudy Dwyer, Agnieszka Sobolewska, Danielle Le Lagadec, Justine Connor, Julie Kahl, Tania Signal, Matthew Browne

**Affiliations:** 1 Central Queensland University, School of Nursing & Midwifery, Building 18, Bruce Highway, Rockhampton, 4702 Australia; 2 Central Queensland Hospital and Health Services, Canning Street, Rockhampton, 4701 Australia

**Keywords:** Early warning systems, Nursing, Compliance, Patient safety, Conceptual framework, Qualitative research, Sociocultural

## Abstract

**Background:**

Early warning systems (EWS) are most effective when clinicians monitor patients’ vital signs and comply with the recommended escalation of care protocols once deterioration is recognised.

**Objectives:**

To explore sociocultural factors influencing acute care clinicians’ compliance with an early warning system commonly used in Queensland public hospitals in Australia.

**Methods:**

This interpretative qualitative study utilised inductive thematic analysis to analyse data collected from semi-structured interviews conducted with 30 acute care clinicians from Queensland, Australia.

**Results:**

This study identified that individuals and teams approached compliance with EWS in the context of 1) the use of EWS for patient monitoring; and 2) the use of EWS for the escalation of patient care. Individual and team compliance with monitoring and escalation processes is facilitated by intra and inter-professional factors such as acceptance and support, clear instruction, inter-disciplinary collaboration and good communication. Noncompliance with EWS can be attributed to intra and inter-professional hierarchy and poor communication.

**Conclusions:**

The overarching organisational context including the hospital’s embedded quality improvement and administrative protocols (training, resources and staffing) impact hospital-wide culture and influence clinicians’ and teams’ compliance or non-compliance with early warning system’s monitoring and escalation processes. Successful adoption of EWS relies on effective and meaningful interactions among multidisciplinary staff.

## Background

Early warning systems (EWSs) are widely used to provide improved detection and management of clinical deterioration for hospitalised patients [1, 2]. Research shows that 85% of severe adverse events are preceded by abnormal physiological signs up to 24 h prior to a serious clinical event [3]. If deterioration is recognised and managed early, complications arising from delays can be reduced, and some serious adverse events can be prevented [4, 5].

The use of EWS can potentially counter the problem of in-hospital adverse events [6]. EWS comprises of two components: recognition, also known as “track and trigger”, and response [7]. Nurses have a crucial role in “tracking”: monitoring and measuring patients’ vital signs and documenting them [1, 2] typically on paper-based observation charts. If the patient’s weighted EWS score reaches a predetermined threshold score for intervention, the nurse is required to “trigger” an escalation process which stipulate actions and response times commensurate with the trigger score [8]. When high level thresholds are met, patients are to be clinically reviewed by a team of specialists, often referred to as Rapid Response Teams (RRT) or Medical Emergency Teams (MET) [3]. Deployment of these specialist teams to the patient’s bedside is the response system of EWS. The specialist teams comprise expert clinicians, who once being called, are mandated to intervene in diagnosing and managing the deteriorating patient [1, 9].

Research shows that acute care clinicians inconsistently comply with EWS protocols and processes, with an international study involving 3000 patient charts revealing the main issues were EWS score accuracy, monitoring frequency and delayed escalation [10]. Further, in an observational study of 441 nurse-patient interactions, Cardona-Morrell et al. [11] found inconsistencies in monitoring and recording of the vital signs in a large urban hospital. A full set of observations were taken in only 6–21% of instances of vital signs monitoring. Guinane et al. [12] conducted a retrospective chart audit of 568 patients and reported that while one in seven patients fulfilled MET criteria, activation of the MET occurred infrequently.

Many factors can impact on compliance with EWS protocols and procedures [7, 10] with Shearer et al. [6] reporting that clinicians’ non-compliance was related to local sociocultural factors and professional hierarchies rather than individuals’ failure of cognition. Interviews were conducted with 83 clinicians involved in failure to activate a response, who were employed in a large organisation where EWS non-compliance was a recognised problem [6]. The most common reason for non-compliance was that the bedside staff felt that the clinical situation was under control in the ward setting [6]. In another study, Kitto et al. [13] found that the reasons for non-compliance included: distinct intra-professional clinical decision-making pathways; differences in nursing and medical cultures; and inter-professional communication barriers [13]. While studies have highlighted staff non-compliance with EWS escalation protocols [6, 13], there is a paucity of research focusing on the sociocultural factors impacting compliance with EWS monitoring protocols.

This study explores the sociocultural factors influencing acute care clinicians’ compliance with the monitoring and escalation component of an early warning system (EWS) in their routine practice pertaining to recognition and response of patient deterioration.

## Methods

### Study design

In order to gain a deep understanding of the topic under examination, this interpretative qualitative study utilised inductive thematic analysis to analyse data collected via semi-structured interviews.

### Subjects and setting

Acute care clinicians: enrolled nurses, registered nurses and medical doctors employed in Queensland’s Public Health Sector were eligible to participate in the study. Eligible participants were required to use the most prevalent EWS used in Queensland, Australia, called Q-ADDS – Queensland Adult Deterioration Detection System [14, 15]. It was important to capture data about the use of EWS from both nurses and doctors as these cohorts engage with the EWS in varying ways. Nurses predominantly use the EWS for documenting vital signs and as a basis for triggering escalation of care in response to EWS calculation of patients’ clinical acuity. Medical doctors engage with the EWS by modifying patient’s vital sign parameters. Modification is required when symptoms related to a patient’s chronic medical condition, for example Chronic Obstructive Pulmonary Disease (COPD), has the potential to repeatedly trigger unnecessary clinical interventions [7]. Participants were recruited using purposive and snowball sampling. The study and its website were promoted via the health service organisation’s internal email system, word-of-mouth, and social media. Participants were encouraged to share the website link with their colleagues.

### Data collection

This study employed semi-structured interview techniques involving open-ended questions (interview guide included as supplementary file) which were developed to elicit participants’ perceptions related to their reasoning and behaviours in complying and non-complying with EWS processes and protocols. Participants were encouraged to provide examples from their own practice and workplace. Nurses were asked questions about their use of EWS when monitoring and escalating care for patients. Medical officers were asked about their use of EWS in their routine practice and when responding to actual or reported patient deterioration. Phone interviews, none longer than 60 min, were conducted by two members of the research team (TF JC) between September and November 2018 and were recorded and transcribed.

The research team met regularly to discuss the progress of data collection and assess the degree to which newly collected data was repeating what was already expressed. Once it was recognised that no new data were present, participant interviews ceased as data saturation had evidently occurred [16].

### Data analysis

Interview transcripts were sent to participants to validate the content [17] and then transcripts and recordings were cross-checked by two researchers before being imported into NVivo 12 software [18] in preparation for analysis. Thematic analysis was conducted following Braun and Clarke’s [19] six-stage framework. After initial familiarisation with data through reading and re-reading of the transcript, codes were generated and then grouped together by the research team as consensus driven themes were identified [20]. The thematic review and theming of definitions were conducted independently by three researchers who discussed and documented findings during group meetings. Next the research team refined and finalised the themes in reference to the interview data. This process, along with the development and maintenance of a transparent audit trail, contributed to the rigour of the study [21].

The resulting themes aligned to two main contexts; clinicians’ use of EWS for monitoring and clinicians’ use of EWS for escalation. Intersecting themes that emerged from the data explained individual and teams’ compliance with EWS within these contexts. When all themes that informed and influenced each other were strategically combined, and overlayed within the organisational context, the resulting conceptual framework became evident. The sociocultural conceptual framework was then mapped graphically to provide a powerful visual representation of the research findings [22].

## Results

A total of 30 participants: 10 medical officers (M = 8; F = 2) and 20 nurses (M = 2; F = 18) participated in the study. Participants’ professional post-graduation experience ranged from 1 to 40 years and were located across rural/remote (*n* = 8; 4 medical officers, 4 nurses), regional (*n* = 16; 4 medical officers, 12 nurses), and metro (*n* = 6; 2 medical officers, 4 nurses) hospital sites.

**Fig. 1:**
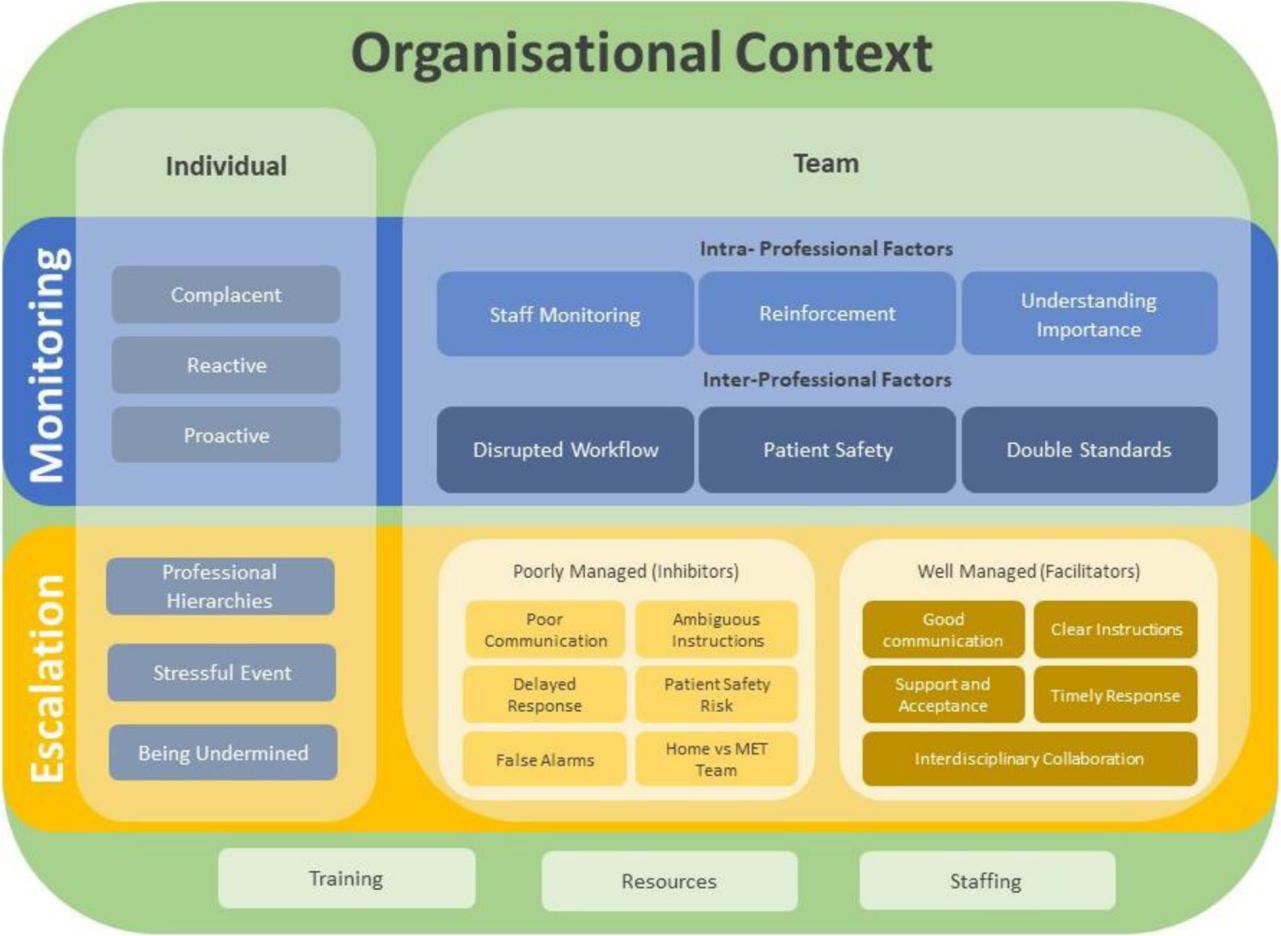
Sociocultural Framework of EWS Compliance. The sociocultural framework for Early Warning System (EWS) compliance, illustrates the individual and team factors that facilitate or inhibit compliance with EWS during monitoring and escalation. Compliance is enacted at an individual and a team level and is impacted by intra and inter-professional factors, and overarchingly, by the organisational context’s quality improvement and administrative protocols

Analysis of data revealed that compliance with EWS was approached in the context of 1) the use of EWS for patient monitoring; and 2) the use of EWS for the escalation of patient care. The identification of these two contexts lead to the development of a conceptual framework as a way to group and explain individual and team behaviours within each context. The sociocultural framework for EWS compliance, presented as Fig. 1, illustrates the individual and team factors that facilitate or inhibit compliance with EWS during monitoring and escalation. Compliance is enacted at an individual and a team level and is impacted by intra and inter-professional factors, and overarchingly, by the organisational context’s quality improvement and administrative protocols.

### The use of EWS for patient monitoring

#### The individual clinician and the complacent, reactive or proactive approach

We identified that the use of EWS for patient monitoring involves nurses collecting and charting patients’ vital signs and medical officers modifying the EWS parameters to avoid unnecessary escalations. Three individual-level approaches to using EWS for patient monitoring emerged: *complacent, reactive, and proactive*. These approaches highlight the differences among the individual clinicians in how they engage with the tool. Please note that throughout the results section we have included each participant’s ID number followed by their professional acronym, either RN (registered nurse) or MO (medical officer).

The complacent approach emerged based on clinicians’ reflections related to their perceptions of their colleagues’ behaviours rather than their own. Incomplete documentation of EWS charts suggestive of incomplete patient observations were common sources of frustration. Participants typically attributed time constraints as reasons for their peers’ non-compliance, especially when increased frequency of observations were required for deteriorating patients. Participants also identified some nurses’ negative attitudes towards EWS. P16_RN. observed: “*they (junior staff) fill it out but aren’t paying attention, they don’t understand what they’ve ticked”. C*omplacency among the senior nurse clinicians was also raised. P10_RN reports: “*there is some open hostility to the form from staff who’ve been around for 20-30 years. They’ll tell you day in and day out that the form’s a load of sh.t and takes away from clinical judgement”*. Complacency among medical officers was evident when they verbalised the modifications but did not chart the articulated changes, or in extreme cases, did not attend the patient when summoned. P15_MO explained that *“some people maybe can’t be bothered to respond because it can be quite tedious, even though you are seeing the numbers there, sometimes it (the EWS Score) is just a guesstimate of the patient’s actual acuity”.*

The prevalence of a reactive approach among junior clinicians emerged. The “reactive approach” denotes the use of EWS in concrete and prescriptive ways, and “reacting” to EWS scores without further investigation. P04_MO observed that “*junior nurses might react rather than do it (escalate care) proactively”*. P14_RN commented: “*the more junior nurses say I have a 5, this is what I need to be doing”.* P08_RN pointed out the usefulness of a score: “*it gives them (junior nurse clinicians) a concise idea of how sick their patient is”*. P15_MO elaborates on how the reactive approach from medical officers’ impacts compliance: “*because it’s a number, it’s been triggered, everyone gets called for no reason just to modify a number. Which can be a waste of everyone’s time...”.*

In contrast, the proactive approach was prevalent among some nurses and medical officers. P22_RN reported using EWS score as ‘a very rough guide’. As a nurse with 28 years of experience, P22_RN asserted*: “this is a blunt tool, I know how to deal with this patient and get the help that I need when I need it….the vital signs are the beginning of your nursing assessment really. If someone has abnormal respiratory rate, then I’m much more concerned about that than what number they’re showing. If I have an asthmatic patient and their respiratory says only 24 but you can hear them wheezing and they’re really tight and there’s not a lot of air moving, then I’m much more concerned about that, and would escalate that much quicker*” . Another experienced nurse P42_RN similarly expressed: “*that even if a patient ‘looks good’ according to the Q-ADDS, she can still escalate care based on her critical thinking and clinical judgement”*. The proactive approach was evident among medical officers when they acknowledged their hesitancy to just write in modifications that would essentially ‘turn off the tool’. P19_MO stated that clinical reviews should not be driven by the pressure to “*modify obs to get a number out of there to stop mediating about that patien*t”. The individual level approaches contribute to the team dynamics or processes in both the intra-professional (nursing team or medical team) and inter-professional teams (comprising of nurses and medical officers) which are considered next.

### The team

#### Intra-professional factors

Nurse participants, both nurse managers and clinicians, identified reinforcement, understanding importance and staff monitoring, as intra-professional team factors contributing to compliance. According to the nurse manager, P32_RN, the reinforcement can be as simple as: “*constantly telling people… [that] if there’s a number then you have to write an intervention”.* P14_RN emphasized that the junior nurse clinicians “*can be influenced by the area or the people they’re working with”*, hence reinforcing good practices on the team level is important. From the nurse manager’s perspective: “*once there’s a proper understanding of the form, we rarely come across the same problems from individual nurses”* (P24_RN). This comment highlights that differences exist in understanding importance of using the form among the nurse clinicians, and that understanding importance can be learnt.

Awareness of being monitored fosters compliance. P39_RN reflected: “*one of our grads, she escalated but she didn’t document any interventions. We actually pulled her in about it and had a big chat. I know for a fact that she’s then spoken to other people about that talk and so now people are on alert”.* It seems that by being monitored, the complacent approach can be changed with a trickle-down effect onto other team members.

#### Inter-professional factors

Nurse participants identified inter-professional double standards in expectations pertaining to EWS compliance. The expectation that EWS documentation is the domain of the nurses, and not the medical officers is apparent. P08_RN reported: “*unless the doctors are prompted by the nurse, they normally don’t write the modifications”*. P17_MO acknowledged: “*we’re often prompted multiple times before we get around to doing it”*.

Nurse participants expressed concern about medical officers’ complacent attitudes and behaviours towards EWS documentation. P37_RN elaborated*: “even if the doctors say yeah, its ok, they need to write yeah that’s ok and they need to follow that up regularly. They don’t do that.”* From the nursing perspective, the inter-professional communication can be characterised by ambiguity, potentially jeopardising the nurse’s EWS compliance. P37_RN emphasised that ultimately the nurse clinicians, rather than medical officers, are held accountable for inconsistent documentation: “*the audit gets done and they’re going, your EWS tool has no modifications on it…there is never an audit into whether the doctor modified the tool correctly”*.

While the nurse clinicians identified medical officers’ non-compliance with documenting modifications as disrupting the workflow, narratives from the medical officers suggest that their non-compliance is more complex than deliberate defiance. P15_MO explained that making modifications requires appropriate expertise: “*my background training is in the ICU so we are a lot more comfortable with modifying physiology parameters... In other departments, say the surgical department, maybe because they are less knowledgeable with medical physiology, they will be more reluctant to modify”*. Other medical participants also indicated that the senior consultants are not readily available to advise junior medical officers regarding modifications.

At times, medical officers encounter tensions between patient safety and EWS compliance. P01_MO elaborated*: “the teams aren’t comfortable with modifying the EWS parameters until the person’s had enough time to be observed...”*. P19_MO further cautioned that clinical reviews should not be driven by the pressure to “*modify obs [observations] to get a number out of there to stop mediating about that patien*t”. Ultimately, patients’ safety must precede EWS procedural compliance.

### The use of EWS for the escalation of patient care

#### The individual clinician

Escalation processes can be either stressful or empowering. Nurse participants commonly described the initiation of escalation of patient care as a stressful event in which the nurse clinician can be undermined. P37_RN reported that medical officers commonly react with “*why are you doing that, stop calling me”*, particularly when being contacted at night from a regional hospital*.* According to P34_RN, EWS is an empowering tool for the nurse clinician: “*it gives you the confidence to say, ‘you need to come review this patient immediately, because they’re scoring a 5’”.* Escalations can be stressful from the medical officers’ perspective. According to P01_MO: “*… the risk is if you modify too many parameters too early, there’s not really any room left and you end up having a real acute deterioration that requires urgent escalation… “.*

### The inter-professional team factors

Inter-disciplinary dynamics are activated during the escalation of patient care. When a patient’s EWS score reaches a predetermined threshold, the nurse is mandated to notify the medical officer. At that stage, additional support and skills are required that fall outside the nursing scope of practice. Escalation processes can either be poorly or well managed, depending on factors such as quality of communication, instructions, level of support and response time.

In a poorly managed escalation of care, the nurse participants identified that professional hierarchies hinder communication and timely responses. P10_RN reflected: “*when they (medical officers) do respond it’s quite often with an eye roll and sometimes a begrudging modification is put in place... And often we’re going off verbal orders, which when it gets to coroner’s court, it doesn’t hold up”.* The ambiguity of medical officers’ instructions can present a professional dilemma for the nurses. P16_RN explained: “*I’ve rung the doctor, they didn’t do the mods (*did not write modifications on the EWS chart*) that they’d written on the chart (*patient notes within the chart*) that they would do. I could do a MET call, but they’ve written in the chart (*patient notes*) that this is their mods”.* P16_RN further reported that “*nurses have to cherry-pick doctors*” to make escalation processes less stressful and more efficient.

The act of making the MET call further exposes the tensions related to the escalation of patient care. P16_RN viewed MET calls as “*behaviour modification”* for the medical officers. P16_RN explained: “*doctors are very bad at including MET call in either the do or don’t section.”* Upon MET’s arrival, “*they [*medical officers*] have to answer to a MET team as to why they hadn’t reviewed the patient in a more timely manner”* (P16_RN). Based on own experience of working in MET, P1_MO elaborated: “*often the nurses have contacted the home team and they haven’t come and done it... If they are not getting anywhere, it can sometimes stimulate it* [stimulate escalation to the MET team] *to action”*. Yet, P1_MO emphasized the value of second opinion: “*sometimes it is good to have someone else who doesn’t know the patient who hasn’t been sitting on it for days”*.

With the MET involvement, a negative feedback loop can ensue related to updating EWS documentation. P16_RN explained: “*the medical teams are aware that the patient’s deteriorating, but they often leave without writing any modification for EWS and then in the next set of observations they* [patient] *score the same thing. If we’re going by the form, we then should be going through the whole process of a MET call again, but verbally, it’s very clear that it’s been seen (*the patient*) and there’s no acute change”*. Here, P16_RN reiterated the challenges of working in an environment which does not strictly comply with the EWS documentation requirements. P16_RN noted that poor communication can extend to the extreme cases: “*they’ll* [medical officers from MET] *write that the patient is dying and not think to tell anyone to stop charting on the EWS”*.

In contrast to poorly managed escalation processes, the well managed approach is characterised by good communication. On-going communication is a feature of positive inter-disciplinary team practice. P40_RN expressed: “*we’ve got great communication with our doctors… we’ll contact them if we think mods need to change”*. P01_MO also elaborated on the interdisciplinary collegiality: “*I am pretty much very compliant with it* [EWS] *and the nurses are very good at notifying teams when patients need review or if there’s something abnorma*l”. Interdisciplinary collegiality fosters a team climate of crises prevention. P42_MO commented: “*even though I don’t technically have to review the patient until the score’s a 4, the nurses are comfortable to come and tell me it’s rising for whatever reason, so it gives me a chance to get on top of it before it is an issue”*.

In the well managed process, the medical officers quickly respond to escalations. P34_RN elaborated: “*before we hit that staff alarm, if we can escalate straight to a senior doctor in ED…my experience is that 99.99% of the time, they will come immediately”.* Another feature of a well-managed escalation of patient care is that junior nurse clinicians are actively supported. P32_RN stated: “*I teach people… ‘you have got a form here that will back you up, it is policy and protocol that you use the EWS form’”.* P05_MO agreed that EWS fosters communication: “*You call up an MO or registrar and ask them to review the patient that has a score of 6 and if any particular parameter is elevated. It makes this communication easier. Less experienced staff might go through the whole story and don’t give the right information. The language becomes easier. ‘A score of 6, you need to see this patient’”.* This comment suggests acceptance and a non-judgemental approach toward the junior nurse clinicians who initiates escalation processes and focuses on the patient’s EWS score rather than their clinical assessment of the patient.

### The Organisational context

The importance of organisational context in shaping EWS compliance or non-compliance emerged. The organisational factors impact on how clinicians engage with and follow EWS protocols. Participants identified training, resources and staffing as the key organisational context factors. P24_RN reflected: “*only when it [*EWS training*] was actually delivered to us did we understand what we’re trying to achieve with it [EWS]”*. P09_MO observed: “… *we had a big advertising/education campaign directed at the nurses to say you must call a MET call, and directed at the doctors to say you must not criticise the nurses when they call a MET call”.* However, training can create unintended consequences. P09_MO reported: *“our MET call numbers went from 2 or 3 a day, to 10 a day and some of them are totally ridiculous”*. Provision of organisational training without the back up support creates additional challenges in responding to patients’ deterioration.

Tension arises between EWS compliance as a labour-intensive process and the availability of resources. P09_MO identified the problem of insufficient staffing: “*you’ll have rural hospitals where there’s one doctor for the whole town who can’t review the patient every two hours”*. Similarly, P16_RN based in a large regional hospital comments: “*they haven’t had stable management in our ED for many years”.* Staffing shortages and the associated instability are a significant challenge. Staffing allocations are also driven by established operational processes rather than the patients’ needs. P34_RN reported: “*we don’t use trends (electronic patient movement software) to estimate nursing hours... whether we end up with 7 or 17 patients, we have the same amount of staffing”.*

Participants in senior positions emphasized the availability of additional resources to support the clinicians’ EWS compliance. Yet, the distribution of these resources varies across locations. P09_MO indicated that: “*a lot of these rural and remote areas you have the benefit of ringing TEMSU (Telehealth Emergency Management Support Unit) with video conferencing and you can ask to speak to a RN if you don’t want to speak to a doctor …”* However the availability of resources varied geographically, with P01_ MO referring to a “*dedicated MET team”*, and P05_MO stating that “*escalations are addressed by the most senior doctor on staff at the time and failing that, the most senior nurse”.* Overall, insufficient resources and staffing were seen as barriers to attaining EWS compliance.

## Discussion

Our study presents a conceptual framework detailing the sociocultural factors facilitating or inhibiting clinicians’ compliance with an EWS. Conceptual frameworks aid in the integration of themes by providing a visual representation of how identified themes ‘fit’ together, how they inform and influence each other, and importantly, how they combine to reveal the bigger, conceptual, picture of what is going on in the area under examination [22]. The development of the conceptual framework aided the identification of the influence the overarching organisation had on the individual and team factors.

The framework (Fig. 1) shows how the two key processes, monitoring and escalation, involve intra-personal responses, working within teams and the overall organisational context. These processes are well documented in the EWS literature [1, 6]. The point of difference in this study is that these processes were examined through a sociocultural, rather than a technical lens. The term sociocultural relates to both social and cultural factors such as beliefs, values and customs present in populations groups such as professions (e.g. nurses or doctors) or workplaces (e.g. wards or hospitals). A ‘social norm’ is an example of a social factor when used to describe the informal understandings that govern behaviour within a population group. One aspect of a population’s culture could be the shared beliefs values and perceptions regarding risks within a workplace and could be referred to as the ‘safety culture’. Importantly, our study highlights the impact that the overarching governance of hospitals, including the embedded quality improvement and availability of resources, have on hospital-wide culture in terms of compliance with EWS.

Our study identified that on the individual level, clinicians approach EWS compliance in one of three ways; complacently, reactively or proactively. These three individual-level approaches highlight different practice implications that ultimately impact patient safety. The complacent approach may place the clinician at risk of missing the vital warning signs of deterioration due to using shortcuts. The prevalence of inconsistent documentation and observations characterising the complacent approach have been documented [11, 23]. In the reactive approach, the clinician may raise false alarms to the annoyance of other clinicians and contribute to alarm fatigue, which is a patient safety concern [24]. In the proactive approach, the clinicians feel confident in their clinical judgement and adopt the wait-and-see approach in dealing with abnormal patient observations. The proactive approach is consistent with prior findings that experienced nurses use a complex interaction of intuition, protocols and clinical judgement to recognize patient deterioration [25, 26]. However, findings of Whyte et al. [27] caution that the amount of experience or knowledge level of experienced clinicians does not consistently translate into effective practice.

The three individual-level approaches suggest the need for ensuring consistency in the intra-professional context. Our study highlighted factors that promote compliance on the intra-professional team level to include reinforcement, staff monitoring and understanding importance. These findings are supported by social learning theories which emphasise that all behaviours are socially learnt through reinforcement and copying others’ behaviours [28, 29]. Acute care research similarly identifies that perceptions about colleagues’ patient safety behaviour and the intra-professional culture influences own behaviours [6, 30].

This study shows that inter-professional team factors influence EWS compliance or non-compliance of the individual clinician. For example, medical officers’ inconsistent documentation of their clinical decisions surrounding the modification of patients’ vital sign parameters, means that nurses cannot always accurately complete patient’s observations. If the nurse completes the observations and scores each vital sign without making allowances for modifications, the score could trigger an unwarranted MET alert. When verbalised modifications have not been appropriately documented, nurses sometimes complete the EWS documentation so that it does not trigger an escalation of care, but in doing so, are employing non-compliant behaviours. This finding highlights that medical officers must exercise caution in order that their inactions, actions or reactions do not delegitimise routine use of EWS observation charts for monitoring, tracking and escalation of care.

Similar to prior research [31, 32], we identified that inter-professional differences, hierarchies and silos can be problematic. Poorly managed escalation processes characterised by poor communication, ambiguous instructions and delayed responses are stressful and risky for patient safety. Conversely, interdisciplinary collaboration, good communication, support and acceptance, clear instructions and timely responses characterise well-managed escalations. The emerging practise implication is that the presence of these inter-professional team factors fosters EWS compliance.

Organisational factors that impact compliance including training, resources (other than staff) and staffing levels were identified. The importance of training when fostering consistent practices across the organisation has been acknowledged previously, with training proving to be particularly important when focusing on compliance with early warning systems [10, 33]. It is well recognised that healthcare delivery relies on the staff to enact processes and behaviours that are legitimised and regulated by the organisation [34, 35]. It is noted, however, that the basic training that legitimises the use of EWS on an organisational level, does not warrant compliance on the ward level [3] and that whilst inter-professional education is thought to facilitate breaking down the professional silos [8], interdisciplinary team work can remain a challenge [31].

In this study, tensions between encouraging escalations of care and servicing high levels of MET calls were identified. This suggests that creating a sustainable change in clinicians’ practice towards proactive compliance with EWS is not a single event but requires an ongoing organisational commitment.

### Limitations

The technical issues including the EWS tool sensitivity were not considered in this study. Instead, the focus was on the sociocultural aspects that emerge through participants’ narratives. We adopted Kitto et al.’s [13] recommendation that EWS implementation research should focus on the sociocultural aspects. The snowball recruitment method gave rise to self-selection of participants. Seidman [36] asserts that interview research explores how the interviewees’ experiences are embedded within the structural and social forces. Representativeness and generalizability are replaced by a focus on the evocation of the participants’ experience [36]. While participants were based in rural, regional and metropolitan hospitals, examination of sociocultural differences across the geographical locations was beyond the scope of the paper. This is a limitation as different hospitals may have unique cultural norms. A limited number of individuals holding different professional roles pose limitations to the generalisability of our findings.

## Conclusion

The analysis of data revealed that the intra and inter-professional factors as well as the organisational context impacts EWS compliance. As this research shows, EWS compliance can be achieved by fostering acceptance and support, good communication, clear instruction and interdisciplinary collaboration. Further research is recommended to use the emerging framework as a basis for developing a sociocultural model of EWS compliance.

## Supplementary information

**Additional file 1.**

## Data Availability

The datasets used and/or analysed during the current study are stored on password protected hard drives and are available from the corresponding author on reasonable request.

## Abbreviations

EWS: Early Warning System
Q-ADDS: Queensland Adult Deterioration Detection System
MET: Medical Emergency Team
ICU: Intensive Care Unit
RRT: Rapid Response Teams
TEMSU: Telehealth Emergency Management Support Unit
CTC: Clinical Team Coordinator
