# Supplementary material for: Developing a sociocultural framework of compliance: an exploration of factors related to the use of early warning systems among acute care clinicians
Source: BMC Health Serv Res. 2020 Aug 11;20:736. doi: 10.1186/s12913-020-05615-6 (PMC7422559; doi:10.1186/s12913-020-05615-6)
Supplement: Supplementary file 1 — Additional file 1. [file 12913_2020_5615_MOESM1_ESM.docx]

The semi-structured interview questions to be used will allow a fuller development of information from the participants. All interviews will begin with the same grand tour questions that aim to elicit rich and detailed information around their compliance using the QADDS:

“Please share with me your experience around factors that influence your compliance with the use of Q-ADDS”

‘Can you explain the behaviours that you employ when you decide to use or not use the Q-ADDS?”

“Can you talk about the reasons you decide to use or not use the Q-ADDS?”
